# Pneumococcal Polysaccharide Abrogates Conjugate-Induced Germinal Center Reaction and Depletes Antibody Secreting Cell Pool, Causing Hyporesponsiveness

**DOI:** 10.1371/journal.pone.0072588

**Published:** 2013-09-12

**Authors:** Stefania P. Bjarnarson, Hreinn Benonisson, Giuseppe Del Giudice, Ingileif Jonsdottir

**Affiliations:** 1 Landspitali, The National University Hospital of Iceland, Department of Immunology, Reykjavik, Iceland; 2 University of Iceland, Faculty of Medicine, Reykjavik, Iceland; 3 Novartis Vaccines and Diagnostics, Siena, Italy; 4 deCODE Genetics, Reykjavik, Iceland; Health Protection Agency, United Kingdom

## Abstract

**Background:**

Plain pneumococcal polysaccharide (PPS) booster administered during second year of life has been shown to cause hyporesponsiveness. We assessed the effects of PPS booster on splenic memory B cell responses and persistence of PPS-specific long-lived plasma cells in the bone marrow (BM).

**Methods:**

Neonatal mice were primed subcutanously (s.c.) or intranasally (i.n.) with pneumococcal conjugate (Pnc1-TT) and the adjuvant LT-K63, and boosted with PPS+LT-K63 or saline 1, 2 or 3 times with 16 day intervals. Seven days after each booster, spleens were removed, germinal centers (GC), IgM^+^, IgG^+^ follicles and PPS-specific antibody secreting cells (AbSC) in spleen and BM enumerated.

**Results:**

PPS booster s.c., but not i.n., compromised the Pnc1-TT-induced PPS-specific Abs by abrogating the Pnc1-TT-induced GC reaction and depleting PPS-specific AbSCs in spleen and limiting their homing to the BM. There was no difference in the frequency of PPS-specific AbSCs in spleen and BM between mice that received 1, 2 or 3 PPS boosters s.c.. Repeated PPS+LT-K63 booster i.n. reduced the frequency of PPS-specific IgG^+^ AbSCs in BM.

**Conclusions:**

PPS booster-induced hyporesponsiveness is caused by abrogation of conjugate-induced GC reaction and depletion of PPS-specific IgG^+^ AbSCs resulting in no homing of new PPS-specific long-lived plasma cells to the BM or survival. These results should be taken into account in design of vaccination schedules where polysaccharides are being considered.

## Introduction

High susceptibility to infectious diseases by polysaccharide (PS) encapsulated bacteria like *Streptococcus pneumoniae* characterizes the neonatal and infant period mainly due to the inability of the neonates and infants to elicit immune response to the PS capsule, a T cell independent type 2 (TI-2) antigen [1], [2]. Neonates become colonized by pneumococci soon after birth, in particular in developing countries, where prevalence of pneumococcal carriage is high [3]. Early colonization and prolonged carriage are believed to contribute to the high incidence and early onset of pneumococcal diseases in developing countries [4]. Furthermore, maternal pneumococcal carriage and younger maternal age are independent risk factors for early onset of pneumococcal carriage in infants in high-risk areas [5].

Hyporesponsiveness, defined as a lower antibody (Ab) level after the second immunization than after the first, has been observed after repeated immunizations with plain pneumococcal PS vaccines (PPV) in infants and toddlers for many of the serotypes [6], [7], as well as in the elderly [8]. Conjugating pneumococcal PS (PPS) to carrier proteins enhances their immunogenicity and renders the immune response T cell dependent (TD) [9]. Pneumococcal conjugate vaccines (PCV), unlike PPV, elicit IgG Abs and immunological memory during infancy [10]. PPS-specific memory after primary PCV series in infants has been demonstrated by plain PPS challenge in the second year, using the anamnestic response and affinity maturation of PPS-specific IgG as surrogate markers for memory [11], [12]. However, PPS administration after PCV priming [13], [14], pneumococcal colonization before or at the time of first infant dose of PCV [15], [16], [17] and previous invasive pneumococcal disease (IPD) [18], [19] have been reported to impair serotype-specific Ab responses to PPV or PCV. The phenomenon of PS-induced hyporesponsiveness is well established [20], but the mechanism is not fully understood. However, PS stimulation of conjugate-induced memory B cells to terminally differentiate into Ab-producing plasma cells without replenishing the memory pool has been suggested [21]. PPV is recommended for elderly and adults 19–64 years of age at high risk of pneumococcal infection [22], whereas PCV is recommended for routine infant vaccinations with 2–3 primary doses before 6 months and a PCV booster 9–15 months of age, as well as for youths aged ≤18 years with underlying medical conditions that increase their risk for pneumococcal disease [23]. The cost of the current PCVs limits their use in resource-poor countries where pneumococcal disease burden is greatest. Other cost-effective alternative schedules have been suggested, both reduction of PCV primary doses and introducing PPV booster instead of PCV [24], [25]. Thus, it is of great importance to understand the precise effects of plain PS on the early life immune system before such policy decisions are made.

Our early life murine model reproduces the main features of infant responses to both plain PPS of serotype 1 (PPS-1) and PCV (PPS-1 conjugated to tetanus toxoid (TT); Pnc1-TT) [26], [27], [28]. We have shown that PPS-1 compromises the ongoing PPS-1-specific Ab response induced by Pnc1-TT priming in neonatal mice, only when administered subcutanously (s.c.), but not intranasally (i.n) [27].

The aim of this study was to determine the effects of single and repeated PPS-1 boosters on conjugate-induced PPS-1- and TT-carrier-specific B cell memory responses in the spleen, and homing of long-lived plasma cells (antibody secreting cells; AbSCs) to the bone marrow (BM) in mice primed with Pnc1-TT as neonates.

The results show that a single dose of plain PPS-1 s.c. is sufficient to completely deplete the PPS-1-specific memory cell and AbSC pool established by pneumococcal conjugate priming in neonatal mice, since PPS-1 abrogates the conjugate-induced germinal center reaction, thus causing hyporesponsiveness.

## Materials and Methods

### Ethics statement

The animal experiments were approved by the Experimental Animal Committee of Iceland (ref. YDL05020034/023/BE), according to the Act on Animal protection nr. 15/1994 (revised Dec. 2008) and Regulations on Animal experimentation nr. 279/2002.

### Mice

NMRI mice (M&B AS, Ry, Denmark) were kept with free access to food and water, with regulated daylight, humidity and temperature. Breeding cages were checked daily and pups kept with the mothers until weaning.

### Vaccine and Adjuvant

PPS of serotypes 1 (PPS-1) and 19F (PPS-19F) was provided and conjugated to tetanus-toxoid (Pnc1-TT, Pnc19F-TT) [29] by Sanofi Pasteur (Marcy l'Etoile, France). LT-K63 was produced and purified [30] by Novartis Vaccines and Diagnostics (Siena, Italy).

### Immunization

Three sets of experiments were performed. In the first set of experiments, presented in the first and second results chapters, neonatal (7 days) mice (8 per group) were immunized s.c. with 0.5 µg Pnc1-TT or Pnc19F-TT (human dose in the PCV Prevenar is 2 µg) w/wo 5.0 µg of LT-K63 in 50 µl saline into the scapular girdle or i.n. in 2× 3.0 µl saline with 30 min interval into the nares. Mice were boosted 16 days later by the same route with saline or 5.0 µg of PPS-1 or PPS-19F (1/5 of a human dose) with 5.0 µg LT-K63 or 0.5 µg conjugate of serotype 1 or 19F with 5.0 µg LT-K63. Control mice received saline at all time-points. The mice were sacrificed 7 days after booster, cells isolated from BM and half of each spleen to numerate PPS-1- and TT-specific IgG^+^ AbSCs by ELISPOT. The other half of each spleen was frozen in Tissue-Tek OCT compound (Sakura, Zouterwoude, The Netherlands) and stored at −70°C until cryosections were cut for immunohistological staining. Serum samples for Ab measurements were obtained 2 days before and 7 days after the booster. In the second set of experiments the long-term effect of PPS-1 booster on PPS-1- and TT-specific IgG^+^ AbSCs in spleen and BM from 7 days to 6 weeks was assessed as presented in the third result chapter. Neonatal (7 days) mice (8 per group and time point) were immunized s.c. with 0.5 µg Pnc1-TT w/wo 5.0 µg of LT-K63 in 50 µl saline or i.n. in 2× 3.0 µl saline (as described above). Mice were boosted 16 days later by the same route with saline or 5.0 µg of PPS-1 with 5.0 µg LT-K63 or with 0.5 µg Pnc1-TT with 5.0 µg LT-K63. Control mice received saline at all time points. Eight mice per group and time point were sacrificed on days 7, 23 and 39 after booster and cells from spleen and BM isolated to numerate PPS-1- and TT-specific IgG^+^ AbSCs by ELISPOT. Serum samples for Ab measurements were obtained 2 days before and 7, 23 and 39 days after booster. In the third set of experiments the effects of repeated PPS-1 boosters s.c. or i.n. on Pnc1-TT-induced PPS-1-specific memory cells in spleen and persistence of long-lived plasma cells in BM was assessed as described in the fourth and fifth results chapters. Neonatal mice (8 per group and time point) were primed with 0.5 µg Pnc1-TT with 5.0 µg LT-K63 in 50 µl saline s.c. or in 2× 3.0 µl saline i.n. (as described above) and boosters of PPS-1+LT-K63 or saline administered by the same routes 1, 2 or 3 times with 16 days interval. Seven days after each administration, at days 7, 23 or 39 after the first booster, PPS-1- and TT-specific IgG^+^ AbSCs were enumerated in spleen and BM. Serum samples for Ab measurements were obtained 2 days before and 7, 23 and 39 days after booster.

### Enumeration of AbSC

PPS-1- and TT-specific AbSCs were enumerated by enzyme-linked immunosorbent spot (ELISPOT) [28]. MultiScreen High protein binding immobilon-P membrane plates (Millipore Corporation, Bedford, MA, USA) were coated with 20 µg/ml PPS-1 (American Type Culture Collection, ATCC, Rockville, MD, USA) or 10 µg/ml TT (Sanofi Pasteur) overnight at 37°C and blocked with RPMI 1640 (Gibco BRL, Life Technologies, Paisley, UK) supplemented with L-glutamin (Gibco BRL), penicillin and streptomycin (Gibco BRL) and 5% foetal calf serum (Gibco BRL). Spleen or BM cells, isolated from immunized mice (10^8^ cells/ml) were serially diluted in complete RPMI 1640 and incubated for 5 h at 37°C. Alkaline phosphate (ALP)-goat-anti-mouse IgG (Southern Biotechnology Associates Inc., Birmingham, AL, USA) was incubated overnight and the reaction developed by 5-bromo-4-chloro-3-indolylphosphate and nitroblue tetrazolium in ALP development buffer (BioRad Labs, Hercules, CA, USA). Spots were counted using a microscope (Zeiss, Oberkochen, Germany) and analyzed with KS ELISPOT (Zeiss).

### Antibody measurements

PPS-1- and TT-specific IgG Abs were measured by enzyme-linked immunosorbent assay (ELISA) [26]. Microplates (MaxiSorp; Nunc AS, Roskilde, Denmark) were coated with 5 µg/ml PPS-1 (ATCC) 5 h at 37°C, or 5 µg/ml TT (Sanofi Pasteur) overnight at 4°C and serum and standard, neutralized by cell wall polysaccharide (Statens Serum Institute, Copenhagen, Denmark), incubated for 2 h, followed by horseradish peroxidase (HRP)-goat anti-mouse Ig (Southern Biotechnology Associates). The reaction was developed by 3,3′,5,5′-tetrametylbenzidine peroxidase substrate (Kirkegaard & Perry Laboratories, Gaithersburg, MD, USA), stopped and read at 450 nm in Titertek Multiscan Plus MK II spectrophotometer (ICN Flow Laboratories, Irvine, UK). Results were expressed as mean log ELISA units (EU)/ml ±SD, calculated from the standard.

### Avidity of PPS and TT Abs

Avidity of IgG Abs was measured by ELISA with a potassium thiocyanate (KSCN) elution step [28]. Results were expressed as avidity index, AI = [M] KSCN displacing 50% of Abs.

### Immunohistochemistry

Spleens frozen in Tissue-Tek OCT (Sakura) were cut into 7 µm cryosections at 4 levels separated by 210 µm, starting 700 µm into the tissue, fixed in acetone for 10 min and stored at −70°C. Four sections/spleen were stained with peanut agglutinin (PNA)-biotin (Vector Laboratories Inc., Burlingame, CA, USA) followed by ALP-conjugated avidin (Mabtech AB, Nacka Strand, Sweden) or TexasRed-avidin (Jackson ImmunoResearch Laboratories Inc., Suffolk, UK) to enumerate GCs, where GCs with immature staining pattern were excluded. Double fluorescent staining was performed with PNA and MOMA-1-FITC (AbD Serotec, Düsseldorf, Germany) that identifies metallophilic marginal macrophages that form a continuous circular lining around the follicles. Sections were also stained with IgM-HRP or IgG-HRP (Southern Biotechnology Associates) to enumerate non-switched and switched B cells. The sections were photographed with a digital camera (AXIOCAM; Zeiss) using a microscope (Zeiss) equipped with ×10 and ×40 objectives and AxioImaging software (Birkerod, Denmark). The average of GCs per follicle was calculated for each spleen.

### Pneumococcal challenge

Six weeks after booster, mice were challenged i.n. with 2.3×10^7^ colony forming units (CFU) of *S. pneumoniae* serotype 1 (ATCC 6301, ATCC) in log-phase resuspended in 50 µl sterile saline, as previously described [26]. After 24 h the mice were sacrificed, blood samples taken from the tail vein and plated on blood agar (Difco Laboratories, Detroit, MI, USA) with Staph/Strep selective supplement containing nalidixic acid and solistin sulphate (Oxoid, Basingstoke, UK) and incubated at 37°C in 5% CO2 overnight. Bacteremia was determined as the number of CFU/ml blood. Lungs were removed, homogenized and diluted to 3 ml saline, serial dilutions plated on blood agar and incubated for 48 h at 37°C under anaerobic conditions. Pneumococcal lung infection was expressed as CFU/ml lung homogenate. Depending on the first dilution used, the detection limits were log 2.2 CFU/ml lung homogenate and log 1.3 CFU/ml blood.

### Statistical analysis

Comparison between groups and time points was performed by Mann Whitney Rank sum test (unpaired data) using GraphPad Prism (GraphPad Software, Inc., La Jolla, CA, USA). A P value of <0.05 was considered statistically significant.

## Results

### Plain PPS-1 booster s.c. depletes PPS-1-specific IgG^+^ AbSCs in spleen induced by neonatal Pnc1-TT-priming

The effects of PPS-1 booster on IgG^+^ AbSCs in spleen and BM and PPS-1 specific antibodies in serum was studied 7 days after priming neonatal mice s.c. or i.n. with Pnc1-TT+LT-K63.

**Figure 1:**
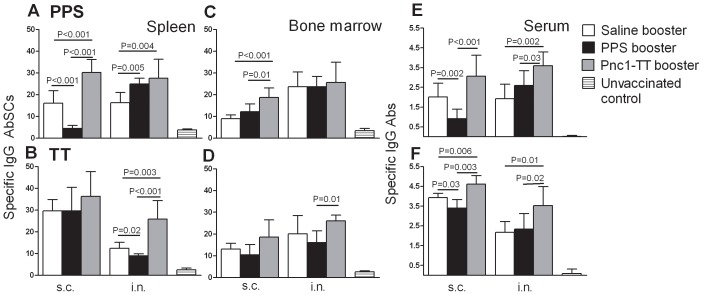
Subcutaneous administration of PPS-1 booster depletes Pnc1-TT-induced PPS-1-specific AbSC pool in the spleen. PPS-1 (upper panels) and TT (lower panels) specific IgG^+^ AbSC measured by ELISPOT in spleen (A and B) and bone marrow (C and D), shown as number of spots (mean±SD) per 10^6^ spleen cells and PPS-1- and TT-specific IgG levels (mean EU/ml±SD) in serum measured by ELISA (E and F). Results are shown for mice boosted by either i.n. or s.c. route with saline (open columns), PPS-1+LT-K63 (black columns), Pnc1-TT+LT-K63 (grey columns) and unvaccinated controls (stribe columns), as indicated. Statistical difference between vaccinated groups is indicated in the graphs. All vaccinated mice had significantly higher frequency of PPS-1 -specific IgG^+^ AbSCs in spleen and BM and also higher serum IgG anti-PPS-1 levels compared to unvaccinated controls (B–F), except mice that received PPS-1 booster s.c. which had comparable frequency of PPS-1-specific IgG^+^ AbSCs in spleen (A). The results shown in A–F are from one of two independent experiments showing comparable results (8 mice per group in each experiment).

Mice primed s.c. with Pnc1-TT and boosted s.c. with PPS-1 had lower frequency of PPS-1-specific IgG^+^ AbSCs in spleen than mice that received saline or Pnc1-TT, reflected in lower serum IgG anti-PPS-1 levels. No difference was observed in the frequency of PPS-1-specific IgG^+^ AbSCs in the BM of mice that received PPS-1 booster compared with saline, but there was a difference compared with Pnc1-TT booster (Figure 1). Mice that received PPS-1 booster i.n. had higher frequency of PPS-1-specific IgG^+^ AbSCs in spleen than mice that received saline, but not in BM (Figure 1). However, no differences were observed between the frequency of PPS-1-specific IgG^+^ AbSCs in the spleen or BM of mice that received PPS-1 or Pnc1-TT boosters. All vaccinated mice had significantly higher frequency of PPS-1-specific IgG^+^ AbSCs in spleen and BM and also higher serum IgG anti-PPS-1 levels compared with unvaccinated control, except mice that received PPS-1 booster s.c., which had comparable frequency of PPS-1-specific IgG^+^ AbSCs in spleen. The depletion of Pnc1-TT-induced PPS-1-specific memory by PPS-1 booster s.c. did not affect the frequency of AbSCs specific for the TT carrier of the vaccine. To investigate whether the interval between the conjugate priming and PPS booster might explain the lack of a response to the PPS-1 booster s.c. at day 16, two groups of neonatal mice were primed with Pnc1-TT and received PPS-1 booster 16 days or 4 weeks later. No IgG response was observed in either group (Figure S1A). Also, different doses of PPS-1 booster (0.5 µg, 2.0 µg or 5.0 µg) were compared, and no response to any of the doses was observed (Figure S1B). Since PPS of serotype 1 is zwitterionic and might therefore be presented within MHC class II and activate T-cells [31], we also tested the effects of PPS booster of a non-zwitterionic PPS following priming with a conjugate of the same serotype. Neonatal mice were primed s.c. with a monovalent pneumococcal conjugate Pnc19F-TT and boosted 16 days later with plain PPS-19F by the s.c. route. As seen for PPS-1, the PPS-19F booster also induced hyporesponsiveness (Figure S1C). The results clearly demonstrate that the PPS-1 booster s.c. compromised the Pnc1-TT-induced PPS-1-specific Abs by depleting PPS-1-specific IgG^+^ AbSCs in spleen, irrespective of dosage, interval between priming and booster administration, and that hyporesponsiveness can be induced by both zwitterionic and non-zwitterionic PPS.

### PPS-1 booster s.c. abrogates the conjugate-induced germinal center reaction

We assessed the effect of PPS-1 booster on Pnc1-TT-induced GC reaction by enumerating GCs and unswitched and Ig-switched B cell follicles, 7 days after boosters with adjuvanted plain or conjugated PPS-1 or saline.

**Figure 2:**
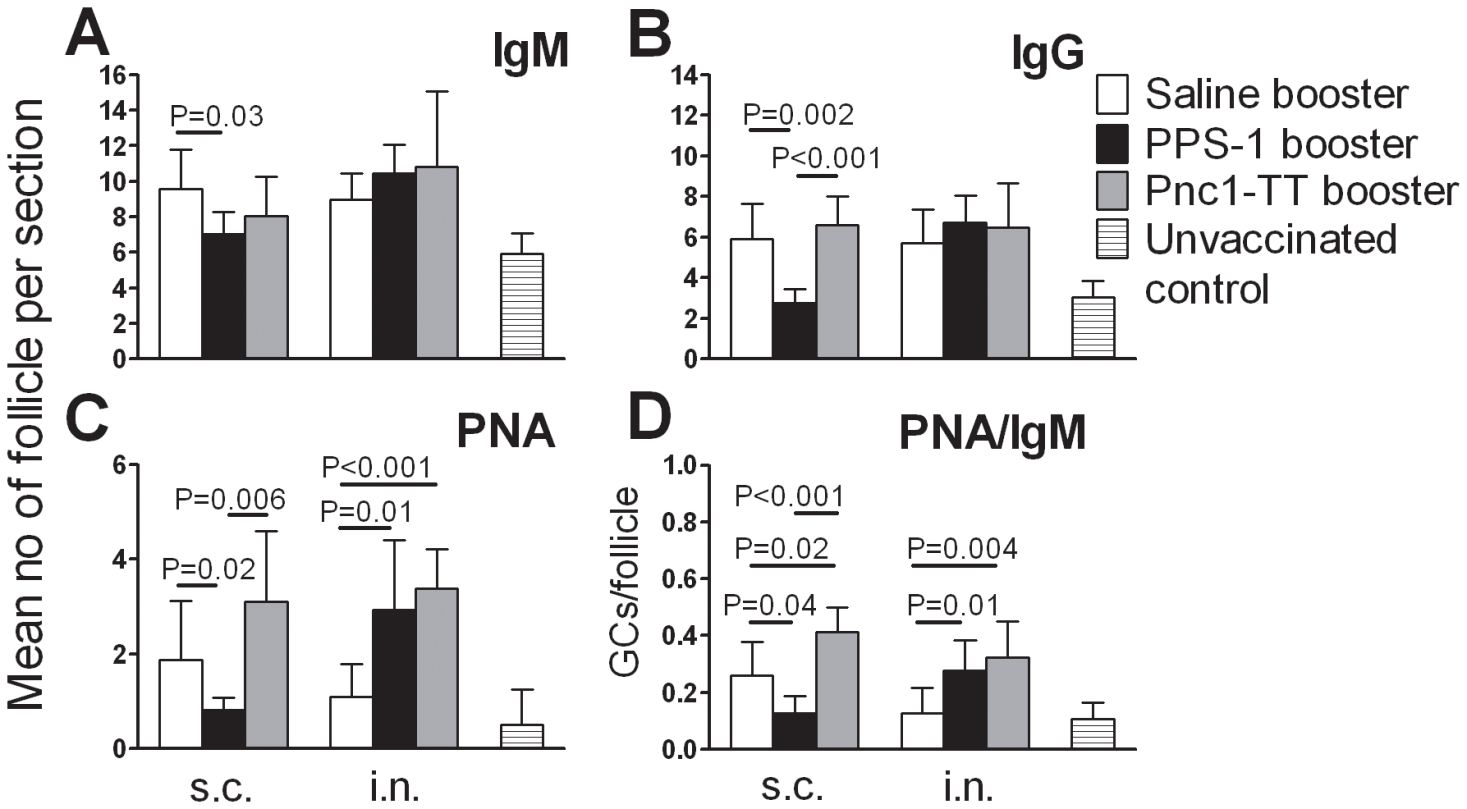
Plain PPS-1 booster abrogates the Pnc1-TT-induced GC reaction in mice primed as neonates. (A–C) Mean number of follicles and (D) ratio of GC/follicle per section in consecutive sections from spleen of mice that received booster by either s.c. (left columns) or i.n. (right columns) route with saline (open columns), PPS-1+LT-K63 (black columns), Pnc1-TT+LT-K63 (grey columns) and unvaccinated controls (stribe columns), as indicated. Spleens were removed 7 days after booster in mice primed as neonates with Pnc1-TT+LT-K63 by the same route as the booster. Half of the spleen was snap frozen, serial cryosection prepared, cutting into 7 µm sections at four levels, starting 700 µm into the tissue and the levels separated by 210 µm. Section from all 4 levels were stained with (A) anti-IgM, (B) anti-IgG, (C) PNA, and results (mean±SD) shown for each group. (D) Mean ratio of GC/follicle was calculated for every spleen at all 4 levels for individual mice and results (mean±SD) shown for each group. Statistical difference between vaccinated groups is stated in the graphs. Results in A–D are from one representative of two independent experiments (8 mice per group) showing comparable results.

**Figure 3:**
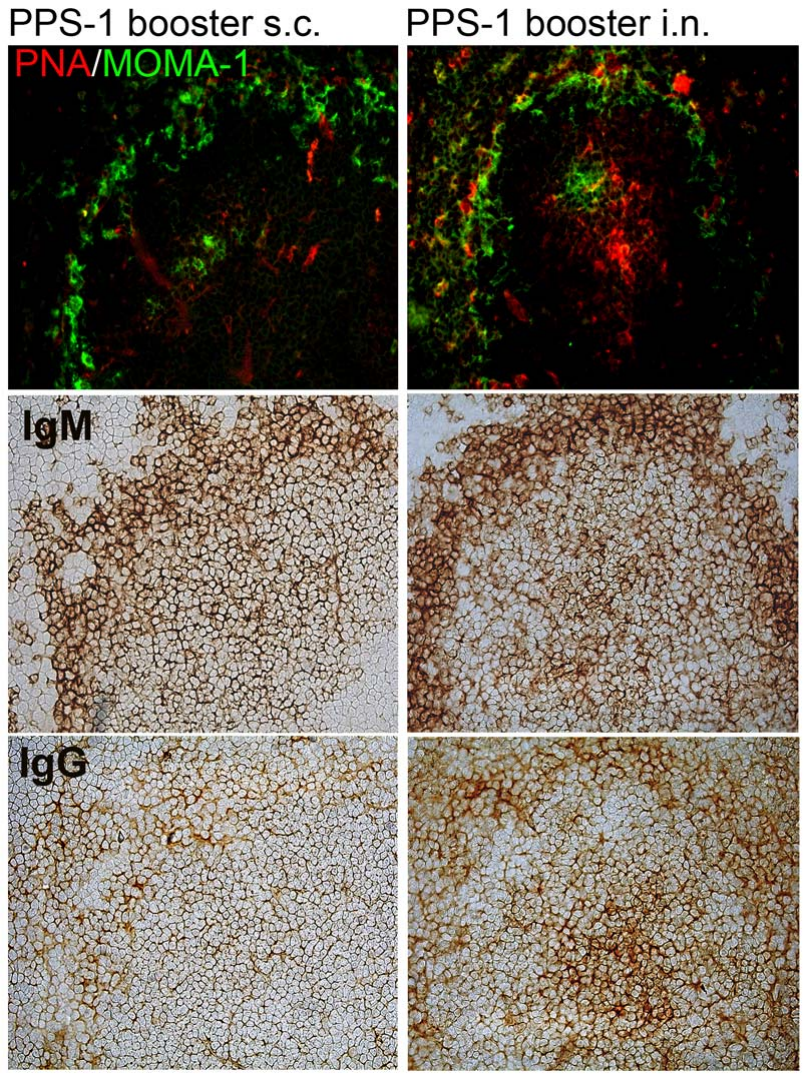
Plain PPS-1 booster s.c. abrogates the Pnc1-TT-induced GC reaction in mice primed as neonates. Active germinal centers in spleen sections were enumerated with PNA staining (upper panels). Double fluorescent staining was performed with PNA and MOMA-1 (metallophilic marginal macrophages) to show the follicular structure (top panel). IgM^+^ and IgG^+^ follicles were identified with anti-IgM (middle panel) and anti-IgG (lower panel) staining, 7 days after booster with 5.0 µg PPS-1 and 5.0 µg LT-K63 s.c. (left) or i.n. (right). Spleen sections, 7 µm, were prepared from four different levels in the spleen, starting 700 µm into the tissue and each level separated by 210 µm. One representative section per group is shown. Results are from one representative of two independent experiments (8 mice per group) showing comparable results.

PPS-1 booster s.c. abrogated the Pnc1-TT-induced GCs, resulting in fewer mature PNA^+^ GCs than after saline booster (Figure 2). In contrast, PPS-1 booster i.n. enhanced GC formation, shown by higher mature PNA^+^ GC numbers in spleen (Figure 2) compared with saline i.n.. Accordingly, higher number of mature PNA^+^ GCs was found after PPS-1 booster i.n. than s.c. (P = 0.002). PPS-1 booster s.c. also reduced unswitched IgM^+^ and switched IgG^+^ follicles compared with saline booster s.c.. In mice that received PPS-1 booster s.c., the PNA staining pattern in spleen differed from that in other immunized mice, as GCs that looked less mature were observed. Also, staining pattern of anti-IgG differed, with less IgG^+^ cells in the marginal zone (MZ) and hardly any in GCs (Figure 3). The ratio of PNA^+^ GCs to IgM^+^ follicles was calculated for each spleen section and used as a marker for Pnc1-TT-induced GCs. The mature GC/follicle ratio was lower after PPS-1 than saline booster s.c., contrary to PPS-1 booster i.n.. Accordingly, the GC/follicle ratio was higher after i.n. than s.c. PPS-1 booster (Figure 2). In contrast to PPS-1 booster s.c., a Pnc1-TT booster resulted in a significant increase in PNA^+^ GCs and the ratio of GCs/IgM^+^ follicles (Figure 2).

These results clearly demonstrate that booster with plain but not conjugated PPS-1 s.c. abrogates the Pnc1-TT-induced GC reaction which parallels with reduced frequency of PPS-1-specific IgG^+^ AbSCs in spleen of mice primed with Pnc1-TT as neonates.

### Long lasting reduction of PPS-1-specific IgG^+^ AbSCs persisted in spleen and bone marrow after PPS-1 booster s.c. in mice primed as neonates with Pnc1-TT

We assessed the long-term effect of PPS-1 booster on PPS-1- and TT-specific IgG^+^ AbSCs in spleen and BM from 7 days to 6 weeks.

**Figure 4:**
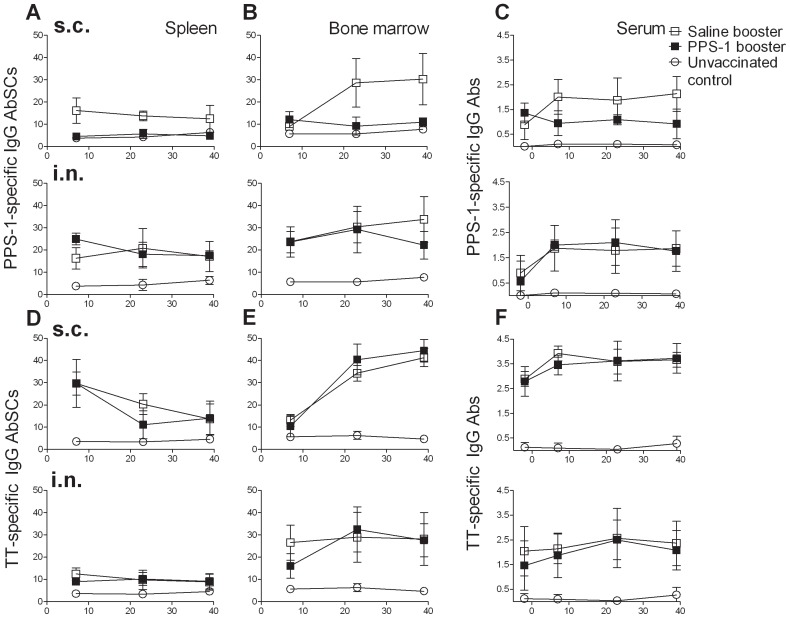
PPS-1 booster depletes Pnc1-TT-induced memory cells in spleen preventing homing of PPS-1-specific AbSCs to BM. PPS-1-specific (A–B) and TT-specific (D–E) IgG^+^ AbSCs, shown as number of spots (mean±SD) per 10^6^ cells, in spleen (A and D) and bone marrow (B and E) measured by ELISPOT, and PPS-1- (C) and TT-specific (F) IgG Abs (mean EU/ml±SD) in serum measured by ELISA, at day 7, 23 and 39 after booster with saline (open squares), PPS-1+LT-K63 (filled squares) or unvaccinated controls (open circles). The results shown are from one of two independent experiments (eight mice/group for each time point) showing comparable results.

**Figure 5:**
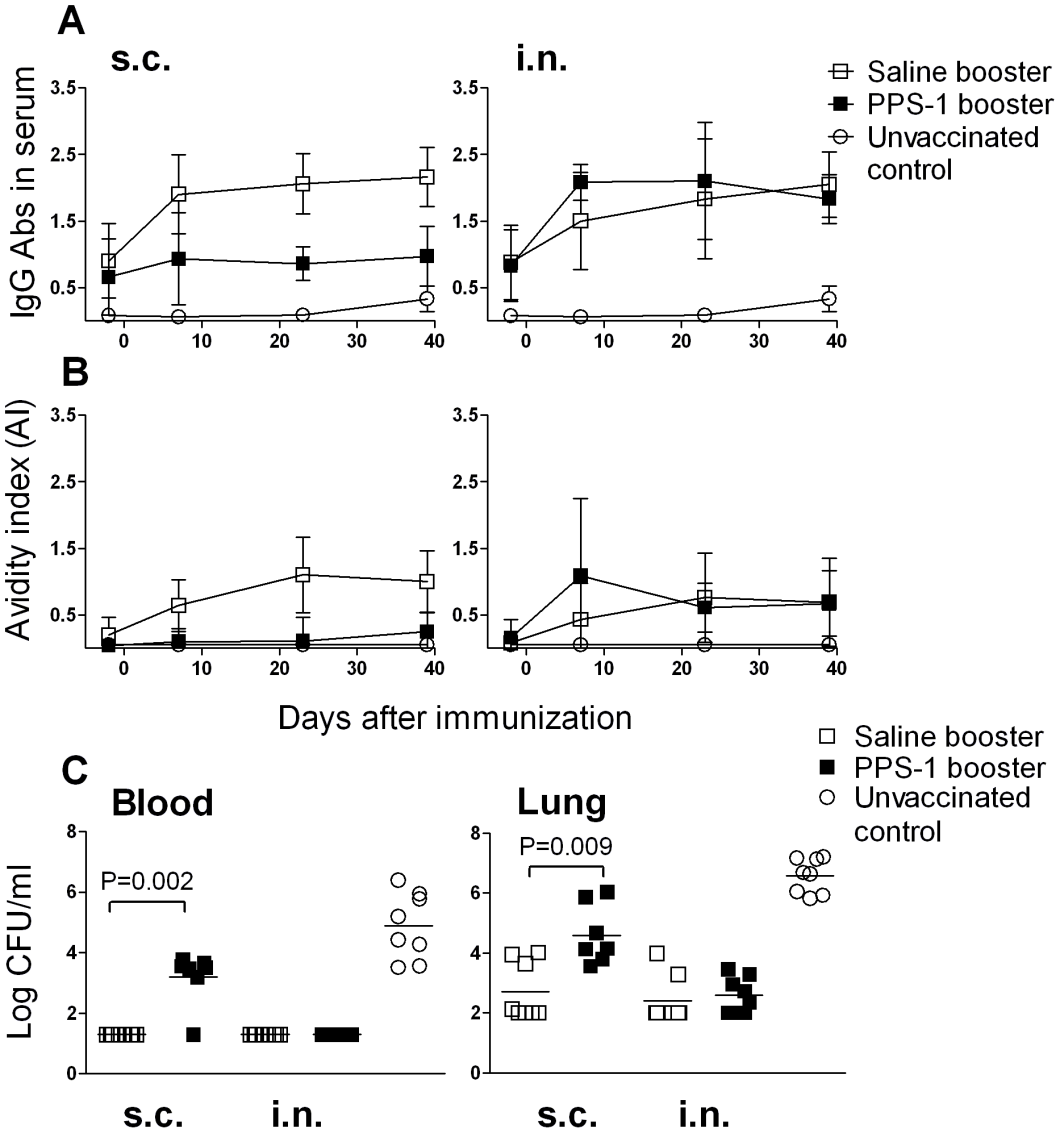
PPS-1 booster abrogation of Pnc1-TT-induced GCs reduces levels, avidity and protective efficacy of PPS-1-specific IgG. PPS-1-specific IgG levels (mean EU/ml±SD) in serum (A) and their avidity index (mean AI±SD) (B) measured by ELISA −2 days before and 7, 23 and 39 days after s.c. (left panels) or i.n. (right panels) booster with saline (open squares), PPS-1+LT-K63 (filled squares) in mice primed with Pnc1-TT as neonates or unvaccinated controls (open circles). Six weeks after the booster the mice were challenged with live *S. pneumoniae* serotype 1 to assess protection against bactermia and lung lung infection [26]. Pneumococcal colony forming units (CFU/ml) in blood (C, left graph) and lungs (C, right graph) 24 h after challenge shown for each mouse (N = 8/group), the median for each group is indicated by a line. Statistical difference in bacteremia or lung infection between vaccinated groups is indicated in the graphs. The results shown are from one of two independent experiments (eight mice/group for each time point) showing comparable results.

As before, the PPS-1 booster s.c. reduced the frequency of Pnc1-TT-induced PPS-1-specific IgG^+^ AbSCs (P = 0.003) at day 7 in spleen compared with saline, but not in BM where the frequency tended to be higher (P = 0.090) (Figure 4). Twenty three days after PPS-1 booster s.c. the frequency was still lower in spleen (P<0.001) and also in BM (P<0.001) than after saline booster s.c. (Figure 4). The reduction persisted at 39 day after PPS-1 booster both in spleen (P<0.001) and BM (P<0.001). These results indicate that the abrogation of Pnc1-TT-induced GC reaction by PPS-1 booster s.c., detected at day 7 in spleen, paralyzed the output of PPS-1-specific IgG^+^ AbSCs from the GCs and depleted the PPS-1-specific IgG^+^ AbSCs formed after Pnc1-TT priming. This resulted in no homing of new AbSCs to the BM, which was reflected in significantly lower IgG anti-PPS-1 levels in serum at all time points (Figure 4). The enhanced induction of PPS-1-specific IgG^+^ AbSCs in spleen after PPS-1 booster i.n. compared with saline at day 7 was diminished at days 23 and 39, and was comparable in BM of these two groups at days 7 and 23. At day 39, however, the frequency of PPS-1-specific IgG^+^ AbSCs in BM was lower after PPS-1 booster i.n. than saline i.n. (P = 0.03). Furthermore, the avidity (Figure 5B) and the protective efficacy (Figure 5C) of the anti-PPS-1 IgG Abs was lower after PPS-1 booster s.c. than saline, contrary to PPS-1 booster i.n. (Figure 5A). The depletion of AbSC by the PPS-1 booster s.c. was restricted to the Pnc1-TT-induced PPS-1-specific IgG^+^ AbSCs, as the frequency of TT-specific AbSCs was unaffected (Figure 4).

Our results demonstrate that a PPS-1 booster given s.c., but not i.n., depleted the Pnc1-TT-induced PPS-1-specific memory cell and AbSC pool in spleen and the reduction persisted until 6 weeks after the booster. No homing of new PPS-1-specific long-lived plasma cells to the BM was detected.

### Depletion of Pnc1-TT-induced PPS-1 specific memory cells by repeated PPS-1 boosters s.c. in mice primed as neonates

We further investigated the effects of repeated (1, 2 or 3) PPS-1 boosters s.c. on Pnc1-TT induced PPS-1-specific memory cells in spleen and persistence of long-lived plasma cells in BM and PPS-1-specific IgG^+^ Abs in serum.

**Figure 6:**
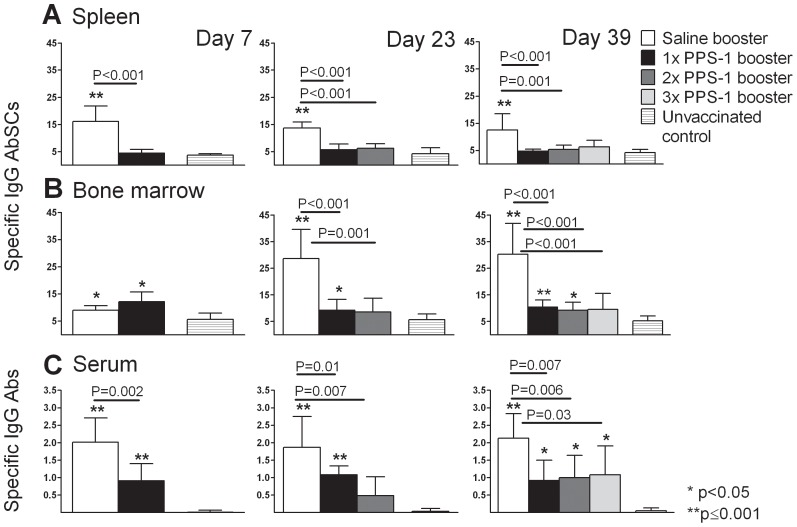
A single PPS-1 booster s.c. is sufficient to completely deplete PPS-1-specific-memory established by neonatal Pnc1-TT-priming. PPS-1-specific IgG^+^ AbSCs, shown as number of spots (mean±SD) per 10^6^ cells, in spleen (A) and bone marrow (B) measured by ELISPOT, and PPS-1-specific IgG Abs (mean EU/ml±SD) in serum (C) measured by ELISA, at day 7, 23 and 39 after mice receiving s.c. booster with saline, PPS-1+LT-K63 or unvaccinated controls. Statistical difference between test groups and unvaccinated controls are indicated as; * P<0.05; ** P≤0.001, and the difference between vaccinated groups is stated in the graphs. The results are shown for one of two independent experiments (eight mice/group for each time point), showing comparable results.

As before, the Pnc1-TT induced PPS-1-specific serum IgG^+^ Abs and IgG^+^ AbSCs were compromised in spleen, but not BM, 7 days after the 1^st^ PPS-1 booster s.c. (Figure 6). Two PPS-1 boosters (23 days after the 1^st^ booster and 7 days after the 2^nd^ booster) reduced the frequency of PPS-1-specific IgG^+^ AbSCs both in spleen and BM and serum IgG anti-PPS-1 levels. Three PPS-1 boosters (39 days after the 1^st^ booster, 23 days after the 2^nd^ booster and 7 days after the 3^rd^ booster) reduced the frequency of PPS-1-specific IgG^+^ AbSCs in spleen and BM and serum IgG anti-PPS-1 levels. There were no differences in the frequency of PPS-1-specific IgG^+^ AbSCs in spleen and BM between one, two or three PPS-1 boosters at days 23 and 39, reflected in comparable serum IgG anti-PPS-1 levels (Figure 6). The detrimental effects of PPS-1 boosters were not observed for Abs or AbSC specific for TT carrier of Pnc1-TT (Figure S2).

These results demonstrate that a single PPS-1 booster s.c. is sufficient to completely deplete the PPS-1-specific memory pool established by Pnc1-TT priming of neonatal mice.

### Repeated i.n. PPS-1 boosters reduce the PPS-1-specific long-lived plasma cell pool in the bone marrow

**Figure 7:**
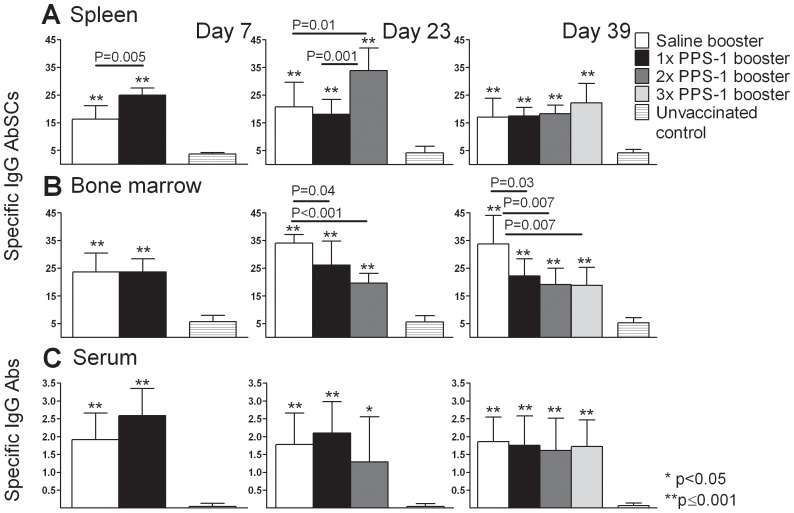
Repeated plain PPS-1 boosters i.n. reduce PPS-1-specific long-lived plasma cell pool in the bone marrow. PPS-1-specific IgG^+^ AbSCs, shown as number of spots (mean±SD) per 10^6^ cells, in spleen (A) and bone marrow (B) measured by ELISPOT, and PPS-1-IgG Abs (mean EU/ml±SD) in serum (C) measured by ELISA, at day 7, 23 and 39 after mice receiving i.n. booster with saline, PPS-1+LT-K63 or unvaccinated controls. Statistical difference between test groups and unvaccinated controls are indicated as; * P<0.05; ** P≤0.001, and the difference between vaccinated groups is stated in the graphs. The results are shown for one of two independent experiments (eight mice/group for each time point), showing comparable results.

Since one i.n. PPS-1 booster did not compromise the Pnc1-TT-induced memory, contrary to the PPS booster s.c., we assessed the effects of repeated PPS boosters i.n. in mice primed with Pnc1-TT as neonates. As previously, the frequency of PPS-1-specific IgG^+^ AbSCs in spleen was higher after PPS-1 compared with saline booster i.n. 7 days after the 1^st^ booster, but AbSCs frequency in BM and serum IgG anti-PPS levels were comparable (Figure 7). At day 23 (7 days after the 2^nd^ booster), two PPS-1 boosters i.n. yielded higher frequency of PPS-1-specific IgG^+^ AbSCs in spleen than saline, but lower frequency in BM. Serum IgG anti-PPS levels were comparable. Thirty nine days after the 1^st^ booster (7 days after the 3^rd^ booster), two or three PPS i.n. boosters resulted in comparable frequencies of PPS-1-specific IgG^+^ AbSCs in the spleen and serum IgG anti-PPS-1 levels as saline administration. However, in the BM the frequency of PPS-1-specific IgG^+^ AbSCs was reduced (Figure 7), whereas TT-specific IgG^+^ AbSCs were comparable in all immunized groups (Figure S3).

These results clearly demonstrate that repeated i.n. PPS-1 boosters compromise the persistence of the PPS-1-specific long-lived plasma cell pool in BM, although less profoundly than s.c. PPS-1 boosters.

## Discussion

This study demonstrates that a single dose of plain pneumococcal polysaccharide s.c. is sufficient to completely deplete the PPS-1-specific memory pool established by pneumococcal conjugate priming in neonatal mice.

The inability of CD21^+^ MZ B cells to localize in the MZ before 2 years of age in humans [32] and 3–4 week in mice [33] as well as low serum C3 levels [34] are considered the major reasons for the lack of PS responsiveness during childhood. However, by conjugating the PS to a protein carrier, the C3 and MZ B cell dependency for the induction of PS-specific IgG response is overcome [35] by T cell help, leading to TD GC induction, production of short- and long-lived plasma cells and memory B cells [36]. This is characterized by immunoglobulin class-switching and extensive somatic hypermutations leading to enhanced Ab affinity for its antigen [37] and a rapid response to re-exposure to the Ag, as accomplished when neonatal mice are primed with Pnc1-TT+LT-K63 [27], [28]. In contrast, plain PS activates B cells through cross-linking of membrane Ig and complement receptor 2 (CR2 or CD21) by C3d-PS complexes [38] limiting the need for direct T cell help. Initiation of Ab response to plain PS depends upon trapping by blood dendritic cells [39] or splenic marginal zone SIGN-R1^+^ MARCO^+^ macrophages [40] that transfer the PS to MZ B cells that rapidly produce protective Abs [41]. Clinical studies have shown a booster response to PPV in toddlers primed with PCV during infancy [7], [42], [43], [44], although the Abs had less functional activity than after PCV booster, both in terms of avidity [14], [43], [45] and opsonic activity [7], [14], [46], [47]. Accordingly, an increased risk of acute lower respiratory infections after PPV booster at 18 months of age was detected in Australian Indigenous children previously primed with 3 doses of PCV during infancy [48]. In our study, PPS-1 did not elicit an anamnestic response, as the frequency of active GCs, PPS-1 specific IgG^+^ AbSCs, IgG Ab levels, avidity and protective efficacy were lower after PPS-1 booster s.c. than saline and Pnc1-TT [26], [27], [28]. The frequency of active GCs and AbSCs, as well as Ab levels, were also significantly higher after Pnc1-TT booster than PPS-1 and saline boosters, as previously shown for Ab levels and their long term persistence [27], further supporting that plain but not conjugated PS booster causes hyporesponsiveness by abrogating the GC reaction and depleting the PS-specific memory cells. Some PSs have zwitterionic charged motifs, including PPS of serotype 1, and may activate CD4^+^ T cells after being processed and presented by MHC class II [31], [49]. However, plain PPS-1 immunization w/wo LT-K63 adjuvant induces no IgG Abs in our neonatal murine model [26]. In agreement with those results, a recent study in humans demonstrated that plain PPS-1 does not elicit memory B cell formation, similar to other PSs [50]. Furthermore, we have shown that a booster s.c. with the non-zwitterionic PPS-19F causes hyporesponsiveness in mice primed with a monovalent pneumococcal conjugate, Pnc19F-TT, reflected in reduced Ab levels compared with a saline administration. Thus, hyporesponsiveness can be induced by both zwitterionic and non-zwitterionic PPS in our neonatal murine model. Furthermore, hyporesponsiveness was induced by PPS-1 booster over a broad dose range and time intervals from priming. The detrimental effect of PPS-1 booster s.c. on the immune response previously induced by Pnc1-TT was reflected in abrogated GC reaction resulting in negligible output of PPS-1-specific plasma cells in the spleen. At that time point, 23 days after Pnc1-TT+LT-K63 priming, the GC reaction in mice that received saline booster by either route is already declining, as the peak of GCs reaction is around day 14 after TT and alum immunization of neonatal mice [51]. Thus, the reduced total number of mature GCs after PPS-1 booster s.c. compared with saline booster s.c. is a strong indicator of the detrimental effects of PPS on the GC reaction and more immature looking GCs appeared after PPS booster s.c.. Accordingly, in humans PPV was recently reported to deplete peripheral serotype-specific memory B cells and the B1b cells in adults 50–70 years of age [52]. We have shown in our neonatal murine model that meningococcal serogroup C polysaccharide (MenC-PS) also induces hyporesponsiveness in mice primed as neonates with a meningococcal C conjugate. The MenC-PS booster induced increased apoptosis of MenC-PS-specific B cells within 8–12 hours, mostly of switched IgG^+^ memory cells [53]. Interestingly, the Pnc1-TT-induced PPS-1-specific long-lived plasma cells that had already homed to their survival niches in the BM were not affected by the PPS-1 booster s.c. and no significant difference was detected in their frequency 7 to 39 days after the booster. But, contrary to mice that received saline as a booster, the frequency PPS-1-specific IgG^+^ AbSCs did not increase, which could be due to abrogation of the GC reaction resulting in no homing of new PPS-1-specific long-lived plasma cells to the BM. Importantly, one dose of PPS-1 booster s.c. was sufficient to deplete the Pnc1-TT-induced memory and AbSCs as each subsequent PPS-1 boosters did not further reduce the frequency of PPS-1-specific IgG^+^ AbSCs in either spleen or BM. Furthermore, we have demonstrated previously that one dose of PPS-1 booster s.c. induces long-lasting abrogation of anamnestic Ab response [27].

Most pathogens enter the body via mucous membranes and vaccines, including conjugate vaccines, administered through mucosal routes can more readily induce mature immune responses in neonatal mice than vaccines administered through systemic routes [54]. However, limited data are available for human infants. Accordingly, i.n. administration of PPS-1 did not abrogate the Pnc1-TT-induced memory, as the frequency of PPS-1-specific IgG^+^ AbSCs in the spleen 7 days after the booster was comparable or higher than in mice that received saline. However, at days 23 and 39 the frequency of PPS-1-specific IgG^+^ AbSCs in the BM was lower in mice that had received PPS-1 i.n. twice or three times than in mice that received saline. Thus, repeated i.n. PPS-1 boosters compromised the long-lived PPS-1-specific plasma cell pool in the BM. Pneumococcal colonization before or at the time of the first infant dose of PCV [15], [16], [17] and previous invasive pneumococcal disease have been shown to impair the serotype-specific Ab responses after previous and to subsequent injections of PCV [19] or PPV [18]. In agreement with the human results we have recently shown that PPV priming of neonatal mice s.c. depletes naïve PPS-specific B cells and impairs the response to subsequent PCV immunization, although the effect varied between serotypes [55]. In the current study we showed that not only parenteral PPS booster, but also repeated exposure to PS at mucosal level, induced hyporesponiveness, although to a lesser extent. In a study using NP-Ficoll as a model TI-2 vaccine, PS-induced immunosuppression upon recall exposure was due to PS-specific IgG Abs [56], also inhibiting stimulation of B cells, but only if added to the culture within the first 24 h [57], suggesting that one potential mechanism of PS-induced hyporesponsivess could be Ag-Ab complexes mediated. Thus, the difference observed between the routes could be due to several factors, including different distribution of cells specialized for antigen uptake, different cell populations at the mucosal inductive sites [58], earlier maturity of mucosal than systemic lymphoid tissues [59], differential organogenesis of lymphoid organs in the neonatal mice [60] and different amount of antigen that reaches the lymph nodes and spleen. We have seen that PPS booster administration intraperitoneally (i.p.) only induced hyporesponsiveness in mice primed as neonates with Pnc1-TT+LT-K63 by either the s.c. or i.p. routes, but not the i.n. route (unpublished data). The cellular mechanisms responsible for PS-induced hyporesponsiveness are not fully elucidated. Our results suggest that PS-induced depletion of PS-specific B cells and apoptosis, primarily of the memory B cell phenotype, is at least one of the mechanisms [53]. PS are large molecules composed of repetitive epitopes, highly resistance to degradation [61] and can persist *in vivo* for a long time [62], [63], [64]. It has been speculated that the existence of a memory B cell pool capable of responding to such persistent Ag needs a suppressive mechanism to prevent their continuous reactivation, overproduction of Abs and that the B cell unresponsiveness elicited by nasopharyngeal carriage, IPD or PPV booster could be a preventative mechanism [15], [19]. Further studies are needed to dissect the exact cellular and molecular mechanisms behind PS-induced hyporesponsiveness. Our results and those of others demonstrate that polysaccharides deplete memory cells and long-lived plasma cells thereby causing hyporesponsiveness in the most susceptible groups targeted by vaccination and warrant reevaluation of all vaccination schedules including polysaccharides.

## Supporting Information

Figure S1 **PPS-1 booster, irrespective of dosage, interval between immunizations and zwitterionic or non- zwitterionic properties, induces PS-hyporesponsiveness.** PPS-1- and -19F-specific IgG levels (mean EU/ml±SD) in serum measured by ELISA, weekly from week 2 to 7 after immunization of neonatal mice with Pnc1-TT+LT-K63 s.c. (A and B) or Pnc19F-TT+LT-K63 s.c. (C) that received a booster with saline (open squares; A, B and C), 0.5 µg of PPS-1+5.0 µg LT-K63 (filled triangles; B), 1.0 µg of PPS-1+5.0 µg LT-K63 (open triangles; B), 5.0 µg of PPS-1+5.0 µg LT-K63 (filled squares; A and B) or 5.0 µg of PPS-19F+5.0 µg LT-K63 (filled squares; C), 0.5 µg of Pnc1-TT (filled circles; A and B) or Pnc19F-TT+5.0 µg LT-K63 (filled circles; C) 16 days later (A left panel, B and C) or 4 weeks later (A right panel). Time of immunization is indicated by arrows.
(TIF)

Figure S2 **The PPS-1 boosters s.c. had no detrimental effects on the frequency of carrier protein-specific AbSCs in spleen and bone marrow.** TT-specific IgG^+^ AbSCs, shown as number of spots (mean±SD) per 10^6^ cells, in spleen (A) and bone marrow (B) measured by ELISPOT, TT-IgG Abs (mean EU/ml±SD) in serum (C) measured by ELISA, at day 7, 23 and 39 after s.c. booster with saline, PPS-1+LT-K63 or unvaccinated control. Statistical difference between test groups and unvaccinated controls is indicated; * P<0.05; ** P≤0.001. The results shown are from one of two independent experiments (eight mice/group for each time point) showing comparable results.
(TIF)

Figure S3 **The frequency of TT-specific AbSCs in spleen and bone marrow was not affected by the PPS-1 boosters i.n..** TT-specific IgG^+^ AbSCs, shown as number of spots (mean±SD) per 10^6^ cells, in spleen (A) and bone marrow (B) measured by ELISPOT, TT-IgG Abs (mean EU/ml±SD) in serum (C) measured by ELISA, at day 7, 23 and 39 after i.n. booster with saline, PPS-1+LT-K63 or unvaccinated control. Statistical difference between test groups and controls is indicated; * P<0.05; ** P≤0.001.The results shown are from one of two independent experiments (eight mice/group for each time point) showing comparable results.
(TIF)
