# Supplementary figures and images for: Pneumococcal Polysaccharide Abrogates Conjugate-Induced Germinal Center Reaction and Depletes Antibody Secreting Cell Pool, Causing Hyporesponsiveness
Source: PLoS One. 2013 Sep 12;8(9):e72588. doi: 10.1371/journal.pone.0072588 (PMC3771989; doi:10.1371/journal.pone.0072588)

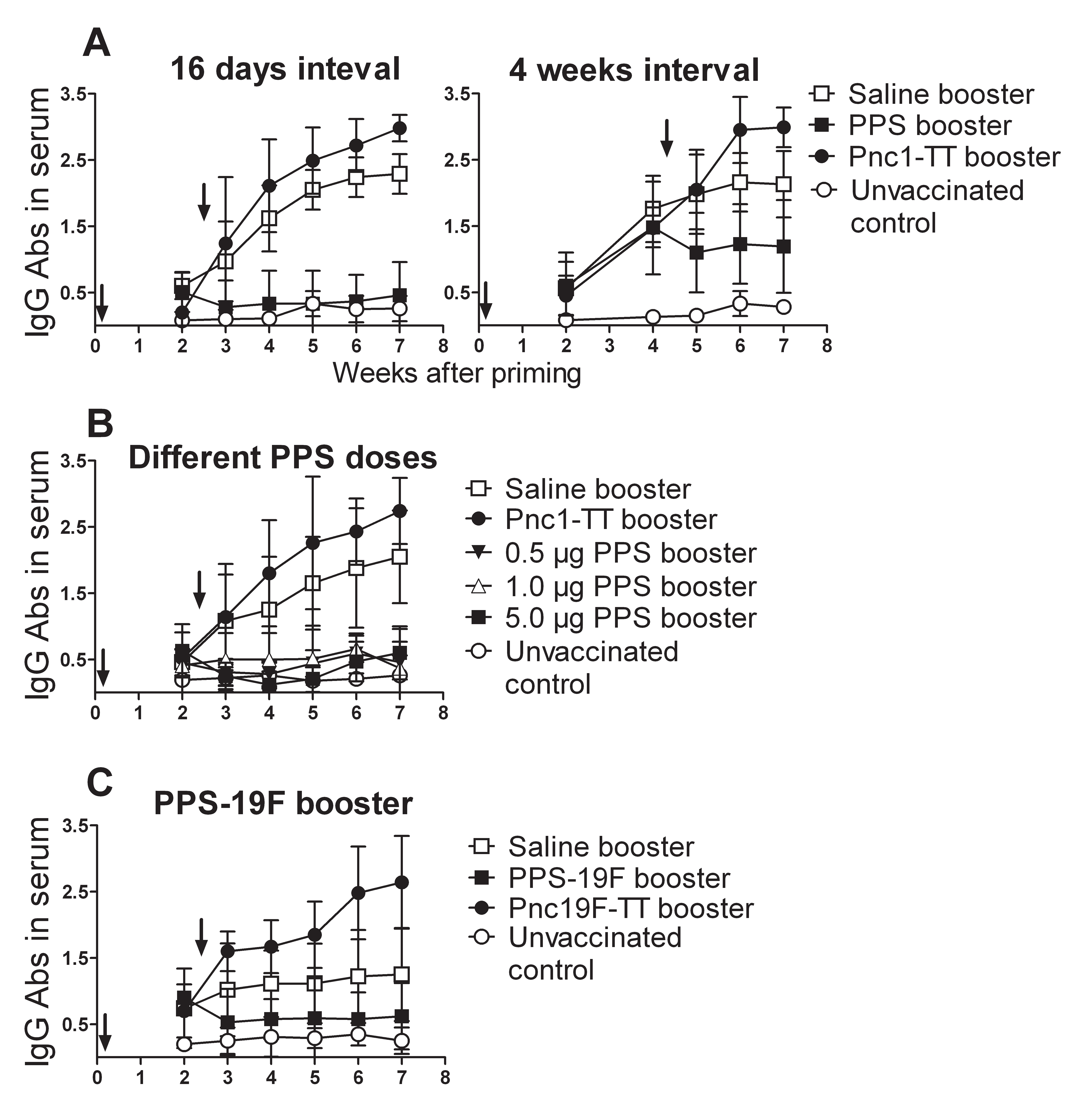

Supplement: Figure S1 — PPS-1 booster, irrespective of dosage, interval between immunizations and zwitterionic or non- zwitterionic properties, induces PS-hyporesponsiveness. PPS-1- and -19F-specific IgG levels (mean EU/ml±SD) in serum measured by ELISA, weekly from week 2 to 7 after immunization of neonatal mice with Pnc1-TT+LT-K63 s.c. (A and B) or Pnc19F-TT+LT-K63 s.c. (C) that received a booster with saline (open squares; A, B and C), 0.5 µg of PPS-1+5.0 µg LT-K63 (filled triangles; B), 1.0 µg of PPS-1+5.0 µg LT-K63 (open triangles; B), 5.0 µg of PPS-1+5.0 µg LT-K63 (filled squares; A and B) or 5.0 µg of PPS-19F+5.0 µg LT-K63 (filled squares; C), 0.5 µg of Pnc1-TT (filled circles; A and B) or Pnc19F-TT+5.0 µg LT-K63 (filled circles; C) 16 days later (A left panel, B and C) or 4 weeks later (A right panel). Time of immunization is indicated by arrows. (TIF) [file pone.0072588.s001.tif]

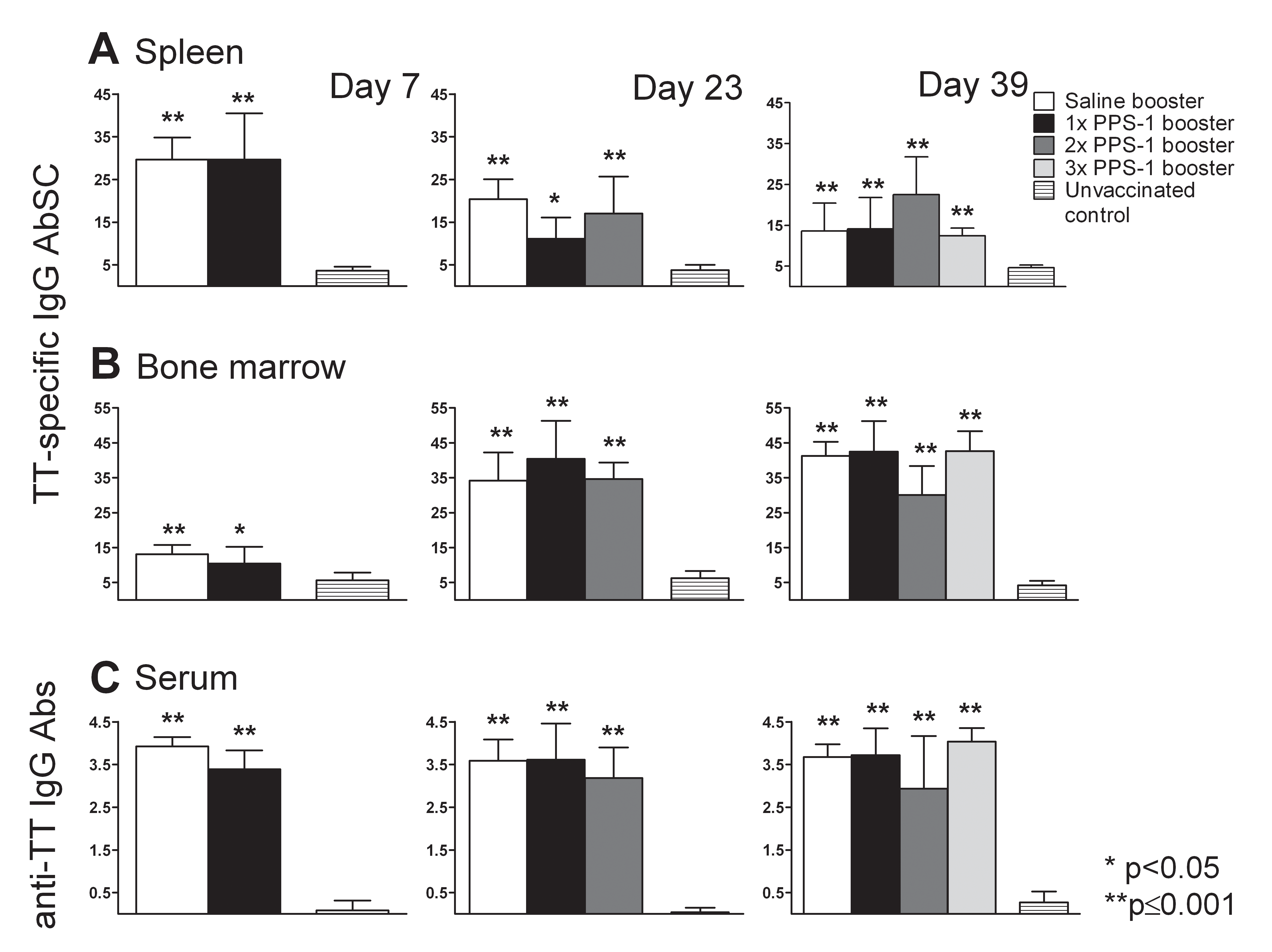

Supplement: Figure S2 — The PPS-1 boosters s.c. had no detrimental effects on the frequency of carrier protein-specific AbSCs in spleen and bone marrow. TT-specific IgG+ AbSCs, shown as number of spots (mean±SD) per 106 cells, in spleen (A) and bone marrow (B) measured by ELISPOT, TT-IgG Abs (mean EU/ml±SD) in serum (C) measured by ELISA, at day 7, 23 and 39 after s.c. booster with saline, PPS-1+LT-K63 or unvaccinated control. Statistical difference between test groups and unvaccinated controls is indicated; * P<0.05; ** P≤0.001. The results shown are from one of two independent experiments (eight mice/group for each time point) showing comparable results. (TIF) [file pone.0072588.s002.tif]

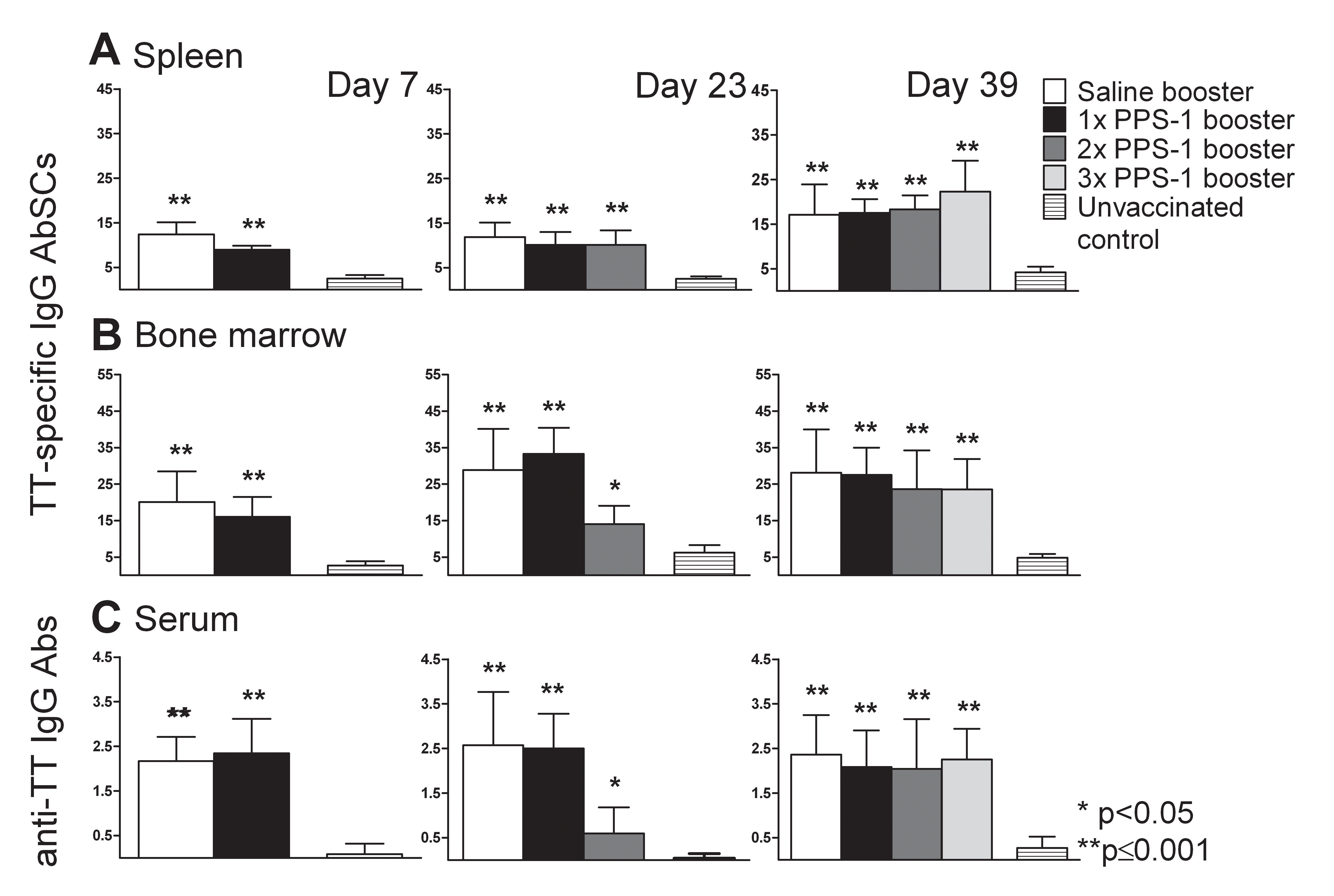

Supplement: Figure S3 — The frequency of TT-specific AbSCs in spleen and bone marrow was not affected by the PPS-1 boosters i.n.. TT-specific IgG+ AbSCs, shown as number of spots (mean±SD) per 106 cells, in spleen (A) and bone marrow (B) measured by ELISPOT, TT-IgG Abs (mean EU/ml±SD) in serum (C) measured by ELISA, at day 7, 23 and 39 after i.n. booster with saline, PPS-1+LT-K63 or unvaccinated control. Statistical difference between test groups and controls is indicated; * P<0.05; ** P≤0.001.The results shown are from one of two independent experiments (eight mice/group for each time point) showing comparable results. (TIF) [file pone.0072588.s003.tif]
